# Directed Transport of CRP Across In Vitro Models of the Blood-Saliva Barrier Strengthens the Feasibility of Salivary CRP as Biomarker for Neonatal Sepsis

**DOI:** 10.3390/pharmaceutics13020256

**Published:** 2021-02-12

**Authors:** Grace C. Lin, Erik Küng, Merima Smajlhodzic, Sandra Domazet, Heinz P. Friedl, Joachim Angerer, Lukas Wisgrill, Angelika Berger, Lynne Bingle, Johannes R. Peham, Winfried Neuhaus

**Affiliations:** 1 Center for Health and Bioresources, Competence Unit Molecular Diagnostics, Austrian Institute of Technology (AIT) GmbH, Giefinggasse 4, 1210 Vienna, Austria; grace.lin@ait.ac.at (G.C.L.); merima.smajlhodzic@ait.ac.at (M.S.); sandra.domazet@ait.ac.at (S.D.); heinz-peter.friedl@ait.ac.at (H.P.F.); joachim.angerer@ait.ac.at (J.A.); johannes.peham@ait.ac.at (J.R.P.); 2 Division of Neonatology, Paediatric Intensive Care & Neuropaediatrics, Department of Paediatrics and Adolescent Medicine, Comprehensive Center for Paediatrics, Medical University of Vienna, Währinger Gürtel 18-20, 1090 Vienna, Austria; erik.kueng@meduniwien.ac.at (E.K.); lukas.wisgrill@meduniwien.ac.at (L.W.); angelika.berger@meduniwien.ac.at (A.B.); 3 School of Clinical Dentistry, University of Sheffield, Broomhall, Sheffield S10 2TG, UK; l.bingle@sheffield.ac.uk

**Keywords:** neonatology, saliva diagnostics, infectious disease, immunology, neonatal sepsis, TR146, HTB-41 (A-253)

## Abstract

C-reactive protein (CRP) is a commonly used serum biomarker for detecting sepsis in neonates. After the onset of sepsis, serial measurements are necessary to monitor disease progression; therefore, a non-invasive detection method is beneficial for neonatal well-being. While some studies have shown a correlation between serum and salivary CRP levels in septic neonates, the causal link behind this correlation remains unclear. To investigate this relationship, CRP was examined in serum and saliva samples from 18 septic neonates and compared with saliva samples from 22 healthy neonates. While the measured blood and saliva concentrations of the septic neonates varied individually, a correlation of CRP levels between serum and saliva samples was observed over time. To clarify the presence of active transport of CRP across the blood–salivary barrier (BSB), transport studies were performed with CRP using in vitro models of oral mucosa and submandibular salivary gland epithelium. The results showed enhanced transport toward saliva in both models, supporting the clinical relevance for salivary CRP as a biomarker. Furthermore, CRP regulated the expression of the receptor for advanced glycation end products (RAGE) and the addition of soluble RAGE during the transport studies indicated a RAGE-dependent transport process for CRP from blood to saliva.

## 1. Introduction

Neonatal sepsis and other neonatal infections account for 0.88% of all disability adjusted life years worldwide, demonstrating similar percentages compared with motor vehicle road injuries (0.98%) and asthma (0.91%) [1]. Neonatal infections are associated with a high mortality [2], brain injury [3], and poor neurodevelopmental outcome in early childhood [4]. Preterm infants are particularly vulnerable to infections and sepsis due to multiple factors such as prematurity, immature host defense mechanisms, frequently used antibiotics combined with invasive interventions, and prolonged stay in the neonatal intensive care unit. Furthermore, clinical signs and symptoms of neonatal sepsis are non-specific, and the differential diagnosis is broad [5]. The adverse outcomes caused by neonatal sepsis in combination with the non-specific symptoms call for early detection, close monitoring, and rapid intervention. Several parameters have been tested for their accuracy in diagnosing neonatal sepsis with clinical studies indicating the applicability of cytokines, presepsin, or S100A2 as biomarkers for detection [6,7,8]. However, the most extensively studied and frequently used laboratory test for diagnosis is based on the serum concentration of C-reactive protein (CRP) of septic neonates [9,10,11]. CRP is an acute phase reactant synthesized in the liver. It has a half-life of 21 h in neonates [12], and it takes 10–12 h for CRP to change significantly after onset of infection [9].

For the detection of neonatal sepsis CRP measurement has a sensitivity of 74–98% and a specificity of 71–94% performed at least 12 h after the onset of symptoms [10]. Serial measurements of CRP 24–48 h after the onset of symptoms increase the sensitivity to 82% and the specificity to 93–96% [13]. These results make CRP the most suitable candidate for monitoring of and routine screening for sepsis in term as well as in preterm neonates. Serum CRP determination is limited by the need for blood sample drawing, especially in preterm children with blood volumes of 80 mL/kg [5], where the volume of blood taken for lab tests should be minimal. Furthermore, every blood sampling carries the risk of infection [14] and is a painful intervention. Therefore, a non-invasive alternative to blood collection would be a great advantage, in particular for preterm and term neonates.

Recent developments in salivary biomarker analysis favor the usage of saliva as a non-invasively collected diagnostic fluid. As the serum/salivary ratio of biomarker concentrations varies between biomarkers [15], sensitive detection methods, such as ELISA or microchips, are necessary. Additionally, knowledge of the correlation between biomarker concentrations in serum and in saliva is essential for salivary diagnostics of diseases.

While previous clinical studies with neonates indicated a positive correlation of salivary CRP with serum CRP concentrations [16,17], a thorough understanding of the transport mechanism from blood to saliva is still lacking. Furthermore, recent studies were inconclusive about the usage of CRP as a salivary biomarker for detection of sepsis in neonates [18,19]. Hence, to understand the detected correlation between saliva and serum concentration of CRP in clinical studies, we collected serum and saliva samples from neonates upon onset of sepsis and during sepsis as well as saliva samples from healthy subjects for comparison. To further investigate the relevance of CRP as a salivary biomarker, we aimed to study the transport of CRP across in vitro models of the blood–saliva barrier (BSB), defined by the epithelial cell layers of the oral mucosa and salivary glands [20]. The human buccal mucosa carcinoma cell line TR146 was chosen as an oral mucosa model, which was recently thoroughly characterized by Lin et al. [21] for its biological barrier properties including expression studies of 96 relevant barrier molecules (cytokeratins, mucins, aquaporins, epithelial-mesenchymal transition (EMT) markers, and tight junction proteins among others) and validation against human buccal biopsy samples. Additionally, the integrity of the paracellular barrier was improved by optimization of the cultivation media and conditions. As a salivary gland model, we used a barrier model based on a single clone of the human submandibular cell line HTB-41, which was previously described on expressions of α-amylase, tight junction proteins and markers specific to different cell types of the salivary gland. Paracellular integrity was verified with transepithelial electrical resistance (TEER) and permeability studies [22]. As both models of the BSB were characterized on the expression and function of transporter proteins as well as tissue specific markers, they were applied for assessment of biomarker transport with ferritin recently and confirmed their suitability for transport studies in biomarker research [22].

Since local inflammation of the oral mucosa may contribute to elevated CRP levels in saliva [23,24], differentiating between transported CRP and locally elevated CRP presents a challenge in the clinic. By utilizing established in vitro models of the BSB, it is possible to study the link between salivary and serum CRP concentrations directly. Moreover, the regulation of the transport mechanism can be examined under pre-set conditions, offering valuable insights in the usability of CRP as a salivary biomarker. Especially the discovery of a transport mechanism for CRP from blood to saliva would offer an explanation for the reported correlation between saliva and blood concentrations in clinical settings. 

## 2. Materials and Methods

### 2.1. Collection of Clinical Samples

This prospective observational part was conducted at the Division of Neonatology, Pediatric Intensive Care and Neuropediatrics at the Medical University Vienna/General Hospital Vienna, a tertiary care academic center, consisting of two separated neonatal intensive care units (NICU, 12 and 10 beds, level IV), two separated neonatal intermediate care units (NIMCU, 2 × 12 beds), and 38 beds on the maternity ward with rooming-in care. This part of the study was approved by the Ethics Committee of the Medical University of Vienna (No. 1265/2016, 24 May 2016) and was performed in two phases. Phase one focused on determining the salivary CRP values in healthy neonates. This was limited to randomly selected neonates born at ≥33 weeks of gestational age and a birthweight of ≥1500 g admitted to the maternity ward without chromosomal aberrations, congenital malformations or inborn metabolic disorders upon obtaining parental informed consent. Saliva sampling was performed at earliest 12 h after birth and earliest 30 min after feeding in clinically healthy neonates. Phase two consisted of evaluation of a correlation between CRP values in saliva and blood. Neonates between 23 and 42 weeks of gestational age admitted to the NICUs or NIMCUs with serum CRP measurements were randomly selected and parental informed consent was obtained. Saliva sampling was performed at the earliest timepoint within 10 h of serum CRP determination and at a minimum of 30 min after feeding without any residues of food present. This time point was chosen based on the time needed for significant changes in CRP values after onset of sepsis of more than 10–12 h and to allow for ideal implementation in the care of critically ill preterm and term infants [9].

In all patients, saliva was collected using the PediaSAL device (Oasis Diagnostic, Vancouver, WA, USA) especially developed for preterm and term infants. This device is designed as “pacifier” with a passive collecting system based on a sponge. This allows to obtain saliva samples of neonates enabling a non-invasive painless sampling with minimal stress. Due to different age and bodyweight of the included neonates, the sponge was used for collection to allow for optimum patient-oriented sampling.

Saliva sampling was most efficient when performed 60–120 min after feeding. To maximize the amount of collected saliva, the following technique proved to be useful: The neonate’s head was placed to the left or right, independent of supine or prone position. A pacifier of according size was offered for 5 min prior to sampling. An increase in the saliva production was achieved by gently stroking the lips with the pacifier or the device. After at least five minutes with the pacifier, the sampling device was placed in the downward cheek of the neonate, where the produced saliva collected. If the amount of saliva was not sufficient, swapping to the pacifier for another 2 min and then back to the sampling device helped. This was sometimes repeated several times. After sampling, the samples were continuously cooled. Saliva was stored within 15 min at −20 °C and subsequently 2 h at −80 °C until transport to the laboratory. Transportation was performed under continuous cooling at −78 °C using dry ice.

### 2.2. Detection of Serum CRP in Clinical Samples

Determination of CRP concentration in serum was performed by the Department of Laboratory Medicine of the Medical University Vienna, Vienna, Austria using the VITROS^®^ 250/350 and VITROS^®^ 5600 System (Ortho-Clinical Diagnostics, Raritan, NJ, USA) or using the Cobas 6000 Chemistry Analyzer (Roche, Basel, Switzerland).

Analysis of CRP using the VITROS^®^ Systems was performed with VITROS^®^ CRP slides (Ortho-Clinical Diagnostics, Raritan, NJ, USA, 23-044-667) and VITROS^®^ Chemistry Products Calibrator Kit 7 (Ortho-Clinical Diagnostics, Raritan, NJ, USA, 1320498). For detection based on an HRP conjugated monoclonal anti-CRP antibody a drop of sample was applied on the slides and measured at wavelength 670 nm upon preparation according to the manufacturer’s instructions. Measurement was performed at a concentration range of 5–90 µg/mL with a limit of detection determined as 2.72 µg/mL by the manufacturer.

For detection of serum CRP concentrations with Cobas 6000 Chemistry Analyzer the CRP-hs reagent kit (Roche, Basel, Switzerland, 4628918190) was used according to the manufacturer’s instruction. Serum was separated from the cells within one hour of collection and stored at −70 °C until measurement. Samples were thawed at room temperature, homogenized, and centrifuged at 2000 rcf for 10 min prior to applying 100 µL for measurement using the c501 module based on a photometric and ion-selective electrode determination. For quality controls an internal pooled serum sample and Precipath U Plus Control (Roche, Basel, Switzerland, 12149443160) was used. Detection limit was determined as 0.15 µg/mL for an analytical measurement range up to 20 µg/mL according to the manufacturer.

CRP concentration in saliva was determined using ELISAs by the Molecular Diagnostic Unit at AIT, the Austrian Institute of Technology, Vienna, Austria, and is described in detail in Section 2.6.

### 2.3. Cell Culture

The human buccal carcinoma cell line, TR146, was purchased from Sigma-Aldrich (St. Louis, MO, USA, 10032305) and cultured in T25 flasks (Greiner, Bio-One GmbH, Kremsmünster, Austria, CELLSTAR^®^, 690175) in Dulbecco’s Modified Eagle Medium (DMEM, Sigma-Aldrich, St. Louis, MO, USA, D5796), supplemented with 10% Fetal Calf Serum (FCS, Sigma-Aldrich, St. Louis, MO, USA, F9665) and 1% Penicillin/Streptomycin (Pen/Strep, Merck, Darmstadt, Germany, A2213) at 37 °C, 5% CO_2_, 95% air atmosphere, and 95% humidity. Media change was performed every 2–3 days and cells were propagated weekly at a cell seeding concentration of 9.33 × 10^3^/cm^2^. For transport studies TR146 were seeded at a cell density of 4.29 × 10^4^/cm^2^ in 300 µL DMEM supplemented with 10% FCS and 1% Pen/Strep (DMEM media) on 24-well ThinCerts (Greiner, Bio-One GmbH, Kremsmünster, Austria, 662641) with 900 µL DMEM media provided on the basolateral compartments in 24-well plates (Greiner, Bio-One GmbH, Kremsmünster, Austria, 662160). To isolate protein, TR146 were seeded at a cell density of 4.29 × 10^4^/cm^2^ in 2 mL DMEM media on 6-well ThinCerts (Greiner, Bio-One GmbH, Kremsmünster, Austria, 657641) with 3.5 mL DMEM media provided on the basolateral side. As soon as cells reached confluency on day 5 or 8 the cultivation condition was switched from submerged to airlift and basolateral DMEM media was changed to DMEM media supplemented with 1% Human Keratinocytes Growth Supplements (HKGS, Thermo Fisher Scientific, Waltham, MA, USA, S0015). Media change was performed every 2–3 days for four weeks. Cells were used from passage 13–38 for the experiments. 

The isolated clone B2 from the human submandibular cell line HTB-41 (ATCC, Virginia, MA, USA) was cultivated in T25 flasks in McCoy’s 5A (Thermo Fisher Scientific, Waltham, MA, USA, 16600082), supplemented with 10% FCS and 1% Pen/Strep, termed as McCoy media, at 37 °C, 5% CO_2_, 95% air atmosphere, and 95% humidity. The isolation was performed previously by single cell experiments using HTB-41 at passage 8 resulting in clone B2 [22]. Cells were propagated weekly at a cell density of 8 × 10^3^ cells/cm^2^ and seeded at a density of 8 × 10^4^/cm^2^ on 24-well ThinCert in 300 µL McCoy media on the apical side, with 900 µL media provided in the basolateral side for transport studies. Clone B2, isolated at AIT, Austrian Institute of Technology, Vienna, Austria was seeded on 6-well ThinCerts in 2 mL McCoy media on the apical side, with 3.5 mL media provided in the basolateral compartment for protein isolation. Media change was performed every 2–3 days until the day of the experiment on day 15 or 16. Cells seeded on 6-well ThinCerts were lysed for protein isolation on day 16. B2 clone was isolated at passage 8 from the parental cell line and subsequently used for experiments at passage 32-48 upon the passage of isolation.

### 2.4. Transport Studies with CRP

On the day of experiment, cell layers of TR146 were washed twice on the apical (300 µL) and basolateral (900 µL) side with Hank’s Balanced Salt Solution (HBSS, Sigma-Aldrich, St. Louis, MO, USA, H6648), while cell layers of the B2 clone were washed twice with basal McCoy media (McCoy’s 5A without supplements). TEER was measured in both models (detailed description in the Supplementary Materials section) to confirm the integrity of the paracellular barrier of the models. Human CRP (Sigma-Aldrich, St. Louis, MO, USA, C4063, 118 kDa, 1 mg/mL in 20 mM TRIS containing 280 mM NaCl) was diluted 1:100 to 10 µg/mL in HBSS or basal McCoy media containing 1% 20 mM TRIS (Roth, Basel, Switzerland, 5429.3, 20 mM) and 280 mM NaCl (Sigma-Aldrich, St. Louis, MO, USA, S7653). HBSS or basal McCoy was replaced with 10 µg/mL CRP on the apical side for transport studies from the apical to the basolateral compartment (A/B) or on the basolateral side for transport studies from the basolateral to apical compartment (B/A).

For transport studies of CRP with the soluble Receptor for Advanced Glycation End products (sRAGE, R&D Systems, Minneapolis, MN, USA, 1145-RG), sRAGE was dissolved in PBS (Thermo Fisher Scientific, Waltham, MA, USA, 14190-094) to 100 µg/mL and subsequently diluted 1:20 in HBSS or basal McCoy containing 1% TRIS, 5% PBS, and 10 µg/mL CRP shortly before the experiment. Media composition was adapted accordingly for all samples and controls during transport studies. Samples on the apical and basolateral side were collected after 24 h and stored at 4 °C until quantification with ELISA. Cell layers of two 24-well ThinCert were lysed with 350 µL RA1 buffer (Machery-Nagel, Düren, Germany, 740961) supplemented with 1% ß—Mercaptoethanol and pooled as one biological sample for RNA isolation.

### 2.5. ELISA for Cell Culture Samples

Transparent high-binding 96-well plates (Corning, Somerville, MA, USA, 3366) were coated with 100 µL 2 µg/mL Human CRP antibody (R&D Systems, Minneapolis, MN, USA, 842676) in PBS [137 mM NaCl (Sigma-Aldrich, St. Louis, MO, USA, S7653), 2.7 mM KCl (Merck, Darmstadt, Germany, 104936), 8.1 mM Na2HPO4 (Merck, Darmstadt, Germany, 106586), 1.5 mM KH2PO4 (Merck, Darmstadt, Germany, 104873), pH 7.3] overnight at 4 °C under orbital shaking conditions sealed with aluminum foil. Prior to usage on the next day, each well was washed three times with 300 µL PBS containing 0.05% Tween 20 (Sigma-Aldrich, St. Louis, MO, USA, P7949; 0.05% Tween 20/PBS) and blocked with 300 µL/well carbonate-bicarbonate buffer (Sigma-Aldrich, St. Louis, MO, USA, C3041) containing 1% BSA (Roth, 0163.2) for 1.5 h at room temperature under orbital shaking conditions sealed with aluminum foil. Consecutively, wells were washed three times with 300 µL/well 0.05% Tween 20/PBS prior to applying the samples.

Samples from the transport studies of the receiving compartment were diluted 1:50; applied CRP or CRP/sRAGE stock solutions 1:2000 in HBSS or basal McCoy containing 1% TRIS for transport studies with CRP and in HBSS or McCoy containing 1% TRIS and 5% PBS for transport studies with CRP and sRAGE. A calibration curve (0, 0.5, 1, 1.25, 2.5, 10, 20 ng/mL) with Human CRP Standard Antigen (R&D Systems, Minneapolis, MN, USA, 842678) was prepared in the respective HBSS or basal McCoy composition used for the dilution of the samples. The diluted samples and the samples for the calibration curve were additionally diluted 1:2 in PBS containing 1% BSA and 20 mM EDTA (Merck, Darmstadt, Germany, 324503) in a non-binding 96-well plate (Corning, Somerville, MA, USA, 3461) and applied as duplicates with 100 µL/well on the previously coated high binding 96-well plate. The plate was incubated for 1 hour at room temperature sealed with aluminium foil under orbital shaking conditions. Afterwards, wells were washed three times with 300 µL/well 0.05% Tween 20/PBS following an incubation step with 100 µL/well of 30 ng/mL detection antibody (R&D Systems, Minneapolis, MN, USA, 842677) diluted in PBS containing 1% BSA (1% BSA/PBS) for 1 h at room temperature sealed with aluminum foil under shaking conditions. After further washing steps, applying 300 µL/well 0.05% Tween 20/PBS for three times, 100 µL/well streptavidin-HRP (R&D Systems, Minneapolis, MN, USA, 890803), diluted 1:200 in 1% BSA/PBS, was added and incubated for 20 min under shaking conditions sealed with aluminum foil at room temperature. After washing three times with 0.05% Tween 20/PBS, 100 µL/well 1-Step^TM^ Ultra TMB-ELISA substrate solution (Thermo Fisher Scientific, Waltham, MA, USA, 34028) was added and the plate was incubated in the dark for 20 min at room temperature.

To stop the reaction 50 µL/well of 1 M HCl (Roth, Karlsruhe, Germany, 6792.1) was added and absorbance was measured at 450 nm with the EnSpire^®^ 2300 Multimode Plate Reader (PerkinElmer, Waltham, MA, US). Permeability coefficient was calculated as apparent permeability values (P_app_) in [cm/s] using the formula shown below (Equation (1)) with c_rec_ as a measured concentration in the receiving side in µg/mL, V_rec_ the volume on the receiving compartment in mL, A as the area in cm^2^, c_0_ as the stock solution in µg/mL, and t as duration in seconds.

(1) $$P_{\mathrm{app}}=\frac{c_{\mathrm{rec}}\;\times \;V_{\mathrm{rec}}}{\mathrm{dt}\;\times \;A\;\times \;c_{0}}$$

### 2.6. ELISA for Saliva Samples

Due to the small saliva volumes of neonates in the range of 30–100 µL, the ELISA method from Section 2.5 had to be adapted to handle 1/5 of the volume. Half-area plates were used (Corning, Somerville, MA, USA, 3690) and the volumes for coating, detection antibody incubation, streptavidin-HRP incubation, and substrate incubation were reduced from 100 µL to 20 µL. Washing and blocking volumes were adjusted from 300 µL to 150 µL. The detection antibody was applied at 100 ng/mL instead of 30 ng/mL. To stop the reaction, 10 µL HCl were used instead of 50 µL. Saliva and standards were diluted 1:2 in 1% BSA/PBS and applied as duplicates using 20 µL/well. All incubation and washing steps were performed under the conditions as described above. Samples were measured with the EnSpire^®^ 2300 Multimode Plate Reader (PerkinElmer, Waltham, MA, USA) at 450 nm as described above. 

### 2.7. Quantitative Real-Time PCR

For RNA isolation of cell lysates, the NucleoSpin RNA Kit (Machery Nagel, Düren, Germany, 740961) was used according to the manufacturer’s instruction and eluted with 40 µL nuclease-free water (Invitrogen, Carlsbad, CA, USA, AM9937). For cDNA synthesis with the Multiscribe Reverse Transcriptase Kit (Thermo Fisher Scientific, Waltham, MA, USA, 4311235) 200 ng RNA were applied for clone B2 while 1000 ng RNA were applied for TR146. Quantitative real-time PCR was performed as triplicates in white 96-well plates (4titue, Dorking, UK, 4ti-0951) with 20 µL reaction volume consisting of 4 µL 1:10 in nuclease-free water diluted cDNA, 2.8 µL 3 µM primer pairs, 3.2 µL nuclease-free water, and 10 µL PowerUp^TM^ Sybr Green Kit (Thermo Fisher Scientific, Waltham, MA, USA, A25742). Primer sequences are shown in Table 1 and were verified with PrimerBLAST [25]. Prior to usage in qPCR, amplicon sizes were evaluated using 2% agarose gels, described in detail in Lin et al. [21], followed by a two-fold dilution series for calculation of primer efficiency performed via qPCR similarly as described by Pfaffl [26]. Primer efficiency was required to be 80–120% to pass for further usage.

The program for qPCR was set for 20 sec at 95 °C for activation, running 40 cycles for 3 s at 95 °C, and 30 s at 60 °C, following a melting stage of 15 s at 95 °C, 1 min at 60 °C, and 15 s at 95 °C using the LightCycler 480 II (Roche, Basel, Switzerland). Data was acquired with the LightCycler480 V1.5 software (Roche, Basel, Switzerland).

For data analysis, ΔCt values were calculated by subtracting Ct vales of the endogenous control from the respective Ct values of the analyzed target. ΔCt values of treated samples were normalized to untreated samples upon exponentiating by 2 as shown with the formula below (Equation (2)).

(2) $$\frac{2^{-{\Delta \mathrm{Ct}}_{\mathrm{treated}}}\;}{2^{-{\Delta \mathrm{Ct}}_{\mathrm{untreated}}}}$$

### 2.8. Western Blotting

On the day of the experiments, cell layers seeded on 6-well ThinCert were washed twice with HBSS (TR146) or basal McCoy (clone B2) on the apical and basolateral side prior to the incubation with HBSS or basal McCoy containing 10 µg/mL CRP or 1% 20 mM TRIS and 280 mM NaCl on the apical and basolateral side for 24 h. Afterwards, cell layers were washed twice with pre-cooled PBS and incubated for 5 min in PBS after the last washing step on ice. For lysis, 50 µL RIPA buffer (50 mM TRIS pH 8.0; 150 mM NaCl, 0.1% SDS, 0.5% sodium-deoxycholate, 1% NP40), supplemented with complete ULTRA protease inhibitor cocktail and PhosphoSTOP minitablet (Roche, Basel, Switzerland, 05892970001 and 04906837001) was added for 30 min on ice. Protein lysates were collected with a cell scraper, and protein concentration was determined with Pierce BCA assay kit (Thermo Fisher, Scientific, Waltham, MA, USA, 23227) according to the manufacturer’s instructions.

Western blotting was performed as described in detail previously [27,28]. In short, 20 µg protein was loaded from each sample, and the primary antibody for RAGE (A-9, Santa Cruz Biotechnology, Santa Cruz, CA, USA, sc-365154, mouse) was applied 1:200–1:300 in 5% nonfat dried milk (ITW reagents, Chicago IL, USA, A0830,0500). Subsequently, the HRP-labeled anti-mouse antibody was applied 1:5000 diluted in 5% nonfat dried milk (Cell Signaling Technology, Danvers, MA, USA, 7076S). For visualization of endogenous control ß-actin, HRP-labeled ß-actin antibody (Sigma-Aldrich, St. Louis, MO, USA, A3854, 7076S) was applied 1:20,000 in 5% nonfat dried milk. Signals were captured with ChemiDoc Imaging system (Bio-Rad, Berkeley, CA, USA), densitometric analysis was performed with ImageLab Software Version 5.2 (Bio-Rad, Berkeley, CA, USA). For analysis of RAGE both detected bands were included in the analysis according to literature describing isoforms of RAGE [29].

### 2.9. Statistical Data Analysis

Results are shown as mean ± SEM unless otherwise indicated. Graphs and statistical analysis were illustrated or performed with SigmaPlot 14.0 (Systat, Jose, CA, USA). Statistical analysis was performed as Mann-Whitney Rank Sum test, Student’s *t*-test, or two-way ANOVA followed by post-hoc Holm-Sidak test with α = 0.05, *p* < 0.05 *, *p* < 0.01 **, *p* < 0.001 ***.

## 3. Results

### 3.1. Analysis of Clinical Samples Propose Individual Correlations between Serum and Salivary CRP Concentrations

In this study, 18 neonates (six females) undergoing sepsis were investigated on their CRP levels in serum and saliva (characteristics of neonates are shown in Table 2).

Nine neonates had one, five neonates had two, two neonates had three, and two neonates had four CRP measurements analyzed over a maximum period of nine days. Sepsis at the time point of sampling was indicated by a threshold of >15 µg/mL CRP in serum.

**Figure 1:**
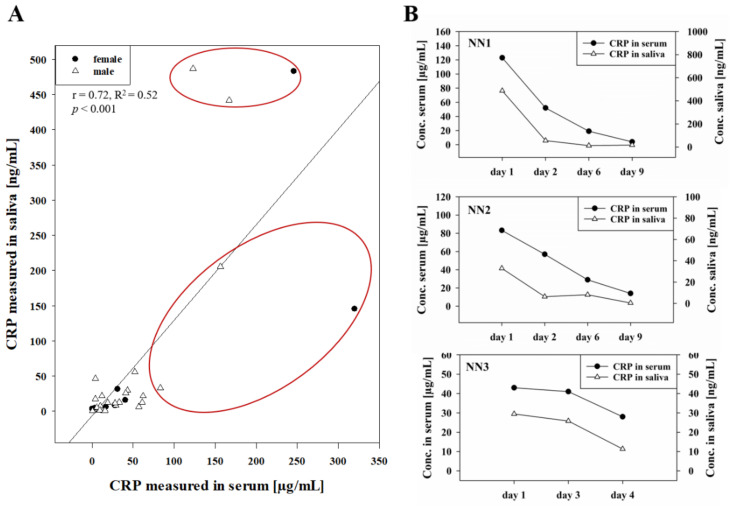
CRP concentration in serum [µg/mL] and corresponding concentration in saliva [ng/mL] of 18 neonates (gestational age at birth 33.10 ± 5.44 weeks, six females) over 1–4 time points (*n* = 33). Serum concentration of CRP was measured with 53.12 ± 72.82 µg/mL (mean ± SD, maximum at 320 µg/mL and minimum at 0.3 µg/mL). Salivary CRP was measured with 65.73 ± 137.70 ng/mL (mean ± SD, maximum at 489 ng/mL and minimum at 0.12 ng/mL). Pearson’s correlation was calculated as r = 0.72 (*p* < 0.001) with *y* = −6.388*x* + 1.358, R^2^ = 0.52. Concentrations indicating severe sepsis (>80 µg/mL CRP in serum) were circled in red (**A**). Time course of measured salivary [ng/mL] and serum [µg/mL] CRP concentration over 4–9 days from three selected neonates (NN1–NN3) with gestational age of 29 + 1, 35 + 0, and 30 + 1 (week + day) (**B**).

Estimated values of CRP in serum and saliva at time point of sepsis varied with 76.40 ± 79.79 µg/mL in serum and 93.70 ± 161.01 ng/mL in saliva (mean ± SD, *n* = 22, median: 4.2 µg/mL and 18.55 ng/mL) resulting in a median ratio between salivary and serum concentration of 1600 (5300 ± 10,100, mean ± SD). However, comparing all measured saliva to serum concentrations (*n* = 33) led to a highly significant correlation between clinical saliva and serum values (Pearson correlation *r* = 0.72, *p* < 0.001, *y* = −6.39*x* + 1.36, R^2^ = 0.52) as shown in Figure 1A. Specifically, an individual correlation of saliva and serum concentrations over time was observed for septic neonates. Exemplary time courses of three neonates over 4 or 9 days are illustrated in Figure 1B.

For comparison of salivary CRP concentrations from septic neonates to baseline saliva levels, samples from 22 healthy neonates (eleven females, Table 3) were measured resulting in significantly lower 14.15 ± 16.63 ng/mL CRP levels in saliva (mean ± SD, median: 6.30 ng/mL, *n* = 22, *p* < 0.05).

### 3.2. Transport of CRP Across BSB In Vitro Models Indicate the Presence of Active Carrier Systems

Clinical data displayed similar progressions of salivary and serum CRP levels for individual neonates over time and indicated a correlation between saliva and serum values. Therefore, we aimed at investigating the transport of CRP between blood and saliva. For this purpose, as a first step 10 µg/mL CRP was added to the oral mucosa epithelium in vitro model on both the apical and basolateral side during preliminary equilibrium studies (Supplementary Materials Figure S1). The data showed a decrease of CRP concentration over time on the basolateral side, indicating a possible transport mechanism in the oral mucosa model.

To study a possibly directed transport in more detail, 10 µg/mL CRP was applied on the basolateral (B/A) or apical side (A/B) for 24 h in the oral mucosa and the salivary gland epithelium model. Significant barrier properties of both in vitro models were confirmed by TEER values of 238.88 ± 54.92 Ω × cm^2^ for the oral mucosa model and 482.70 ± 25.25 Ω × cm^2^ for the salivary gland epithelium model, respectively (raw data: Oral mucosa model: Cells 1015.63 ± 163.45 Ω, blanks 304.67 ± 8.41 Ω; Salivary gland model: Cells 1738.92 ± 74.60 Ω, blanks 302. 33 ± 2.59 Ω).

**Figure 2:**
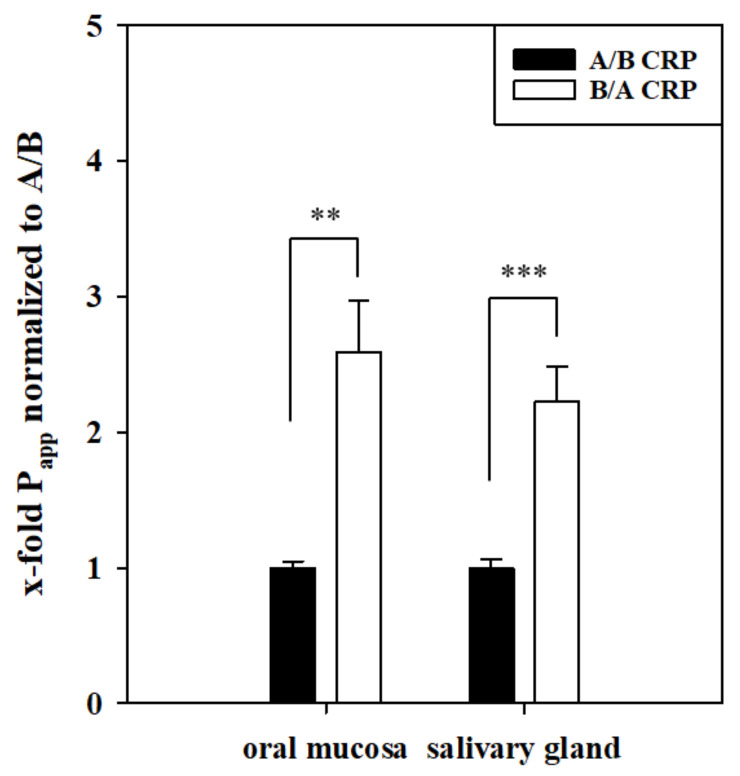
Apparent permeability (P_app_) of 10 µg/mL CRP applied on the apical (A/B) or basolateral (B/A) side for 24 h in the oral mucosa model, based on TR146 cells, and salivary gland model, based on clone B2 of HTB-41 cells. Results calculated as x-fold referred to P_app_ A/B, shown as mean ± SEM after referring to P_app_ values for A/B of each individual experiment of in total three independent experiments (N = 11–13). Statistical analysis was performed as Student’s *t*-test with α = 0.05, *p* < 0.01 **, *p* < 0.001 ***.

Apparent permeability P_app_ values for the oral mucosa model resulted in a 2.58 ± 0.39-fold higher permeability of CRP from B/A upon normalization to P_app_ values from A/B (1.00 ± 0.05, *p* < 0.01; A/B P_app_: 0.11 ± 0.028 × 10^−6^ cm/s, B/A P_app_: 0.21 ± 0.058 × 10^−6^ cm/s), shown in Figure 2. On the other hand, applying 10 µg/mL CRP in the salivary gland model led to a significantly higher permeability from B/A with 2.23 ± 0.26-fold upon normalization to Papp values from A/B (1.00 ± 0.06, *p* < 0.001; A/B P_app_: 0.013 ± 0.001 × 10^−6^ cm/s, B/A P_app_: 0.029 ± 0.003 × 10^−6^ cm/s). In summary, these data clearly indicated the presence of a directed transport mechanism for CRP across the in vitro models of the BSB.

### 3.3. CRP Treatment Modifies the Expression of Receptor for Advanced Glycation Endproducts (RAGE) In Vitro

Previous literature indicated the involvement of RAGE in inflammatory response upon treating endothelial progenitor cells with CRP [30], followed by a subsequent study describing the correlating levels of RAGE and CRP in serum [31].

To examine a possible involvement and regulation of RAGE by CRP, cell samples from the transport studies were analyzed and compared to untreated cell samples at the mRNA and protein level. As a result, the mRNA regulation showed a similar tendency as the P_app_ values described above. 

**Figure 3:**
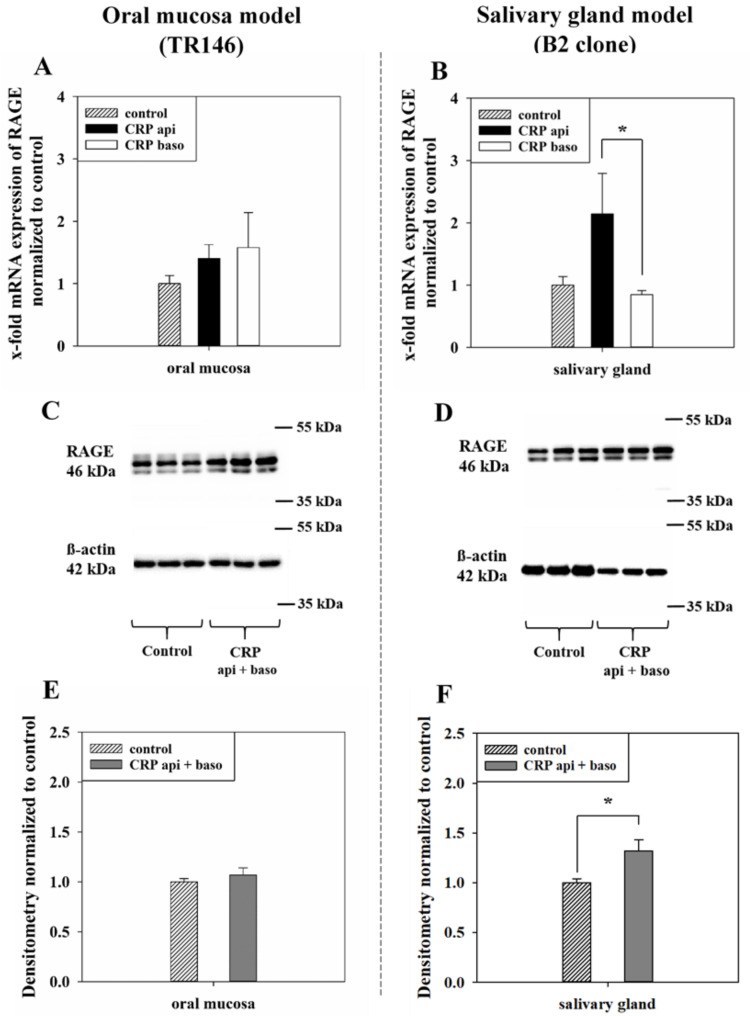
Messenger RNA expression of RAGE of the oral mucosa model (TR146, **A**) and salivary gland model (B2 clone, **B**) after applying CRP on the apical (api) or basolateral (baso) side for 24 h, Ct values were referred to the endogenous control (18sRNA for oral mucosa or PPIA for salivary gland) and normalized to untreated control samples. Results shown as mean ± SEM of two–three independent experiments (N = 4–7). Statistical analysis was performed as one-way ANOVA on ranks with α = 0.05, * *p* < 0.05. Representative western blots of RAGE and ß-actin of the oral mucosa model (TR146, **C**) and the salivary gland model (B2 clone, **D**) after treatment with 10 µg/mL CRP for 24 h on the apical and basolateral side, compared to control samples. Densitometric values of western blots from two—three independent experiments (N= 3) for the oral mucosa model (TR146, **E**) and salivary gland model (B2 clone, **F**) shown as mean ± SEM. Statistical analysis was performed as Student’s *t*-test, with α = 0.05, *p* < 0.05 *.

In the oral mucosa model the addition of CRP resulted in no statistically significant regulation of the mRNA expression of RAGE, only a tendency for increased RAGE expression was observed (1.58 ± 0.56-fold (B/A), 1.41 ± 0.22-fold (A/B), *p* = 0.56). On the contrary, applying CRP in the salivary gland model on the basolateral (B/A) side led to a significant downregulation to 0.85 ± 0.06-fold for RAGE compared to an upregulation of 2.14 ± 0.65-fold upon applying CRP on the apical (A/B) side (Figure 3A,B).

At the protein level (Figure 3C,D), the exposure to CRP on both the apical and basolateral side led to a slight upregulation of RAGE in the oral mucosa model (1.07 ± 0.07, mean ± SEM) compared to control samples (1.00 ± 0.03, mean ± SEM). In the salivary gland model exposure to CRP on the apical and basolateral side led to a significant upregulation of 1.32 ± 0.11-fold (mean ± SD, *p* < 0.05) compared to control samples with 1.00 ± 0.04 (mean ± SEM) (Figure 3E,F).

### 3.4. Soluble RAGE Alters the Transport of CRP In Vitro

To investigate the role of RAGE on the transport of CRP, transport studies with 5 µg/mL soluble RAGE (sRAGE) and 10 µg/mL CRP on the basolateral or apical side were performed and compared to the permeability of CRP alone in both models for each experiment. In the oral mucosa model, transport of CRP from B/A showed a 2.22 ± 0.21-fold higher permeability compared to A/B (1 ± 0.29, *p* < 0.01, P_app_: A/B 0.12 ± 0.017 × 10^−6^ cm/s, B/A 0.22 ± 0.030 × 10^−6^ cm/s), similar to above. Upon normalization to P_app_ values from A/B, the addition of sRAGE led to a similar apparent permeability value of CRP from A/B (0.95 ± 0.08, 0.11 ± 0.0098 × 10^−6^ cm/s), but decreased the transport of CRP from B/A significantly to 1.50 ± 0.08-fold (0.19 ± 0.020 × 10^−6^ cm/s, *p* < 0.001) in comparison to transport of CRP from B/A without addition of sRAGE.

**Figure 4:**
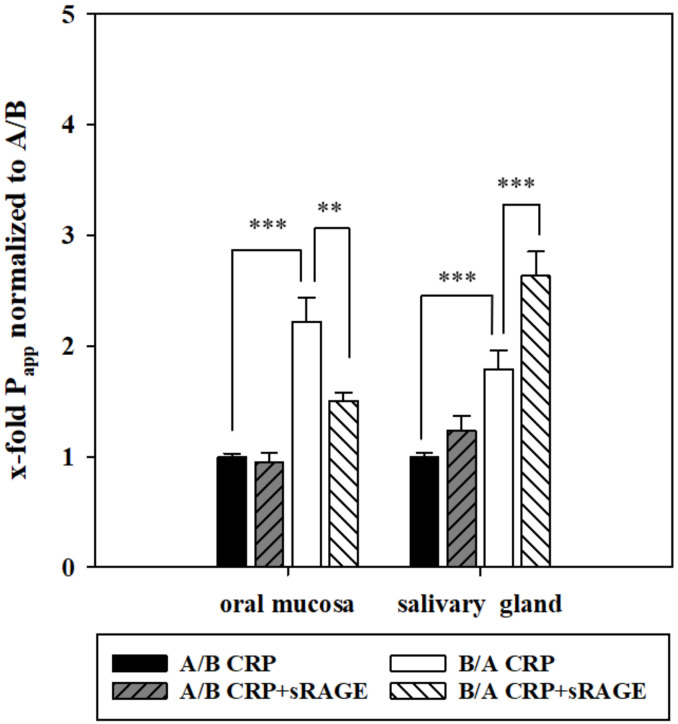
Apparent permeability (P_app_) values of the oral mucosa and salivary gland model upon applying 10 µg/mL CRP or 10 µg/mL CRP + 5 µg/mL sRAGE on the apical (A/B) or basolateral (B/A) side for 24 h. Results shown as mean ± SEM of two–six independent experiments (N = 7–24) upon normalization to P_app_ CRP values from A/B. Outliers were excluded applying Grubb’s test. Statistical analysis was performed as two-way ANOVA, post-hoc Holm-Sidak test with α = 0.05, *p* < 0.01 **, *p* < 0.001 ***.

In the salivary gland model, addition of sRAGE led to a non-significant, slightly increased permeability of CRP from A/B (1.26 ± 0.11-fold, 0.026 ± 0.002 × 10^−6^ cm/s) compared to CRP alone from A/B (1.00 ± 0.034-fold, 0.022 ± 0.009 × 10^−6^ cm/s, *p* = 0.44). In case of the transport B/A, addition of sRAGE resulted in a significantly upregulated permeability of CRP (2.63 ± 0.22-fold normalized to P_app_ A/B CRP (0.06 ± 0.003 × 10^−6^ cm/s)) compared to CRP alone (1.79 ± 0.017-fold, 0.03 ± 0.003 × 10^−6^ cm/s) (Figure 4).

## 4. Discussion

CRP is widely used as an inflammatory biomarker in serum and associated with cardiovascular diseases at levels >10 µg/mL [32]. Recently, an elevated CRP baseline was also linked to a faster progression of Amyotrophic Lateral Sclerosis (ALS) compared to patients with lower CRP baselines [33]. Additionally, higher CRP levels were measured in patients suffering from schizophrenia, possibly linked to inflammation in the pathogenesis of schizophrenia [34]. Furthermore, elevated CRP levels were also determined in patients with SARS-Cov-2 [35], demonstrating its clinical relevance for a broad range of diseases.

First clinical studies using CRP as biomarker to detect bacterial infections were performed in the late 1980s, and CRP was used in the 1990s for detection of septic neonates showing promising clinical usage of CRP for diagnosis of neonatal sepsis [36,37,38,39]. The most recent study correlating neonatal sepsis with elevated levels of CRP and IL-6 was published by Cortes et al. and demonstrated that CRP could be a useful biomarker in combination with other inflammatory biomarkers, especially for late-onset neonatal sepsis [40]. Recently, 14 clinical studies were summarized by Pay and Shaw [41] ) showing a correlation of salivary CRP and serum CRP levels with R^2^ of 0.53 ± 0.23, comparable to the result of our study with a R^2^ value of 0.52 (Figure 1A). Patients with samples drawn at several time points (Figure 1B) in this study showed a median ratio between salivary and serum concentration of 1600 (5300 ± 10,100, mean ± SD) similar to the ratio found in a clinical study with 61 healthy volunteers by Ouellet-Morin et al. [23] reporting a ratio of 1633. This ratio was below the assumed factor of 10,000 described by Pay and Shaw [41].

However, Pay and Shaw [41] also stressed the necessity to understand the correlation of salivary and serum concentrations as local oral inflammation cannot be excluded in clinical set-ups. Even though clinical studies linked salivary CRP positively as a marker for metabolic syndrome [42], detection of obesity in children [43], or pneumonia in neonates [17], a study published in 2019 with 37 healthy young males showed no clear correlation between salivary and serum concentrations of CRP [44]. Additionally, even though CRP is commonly used as a marker for neonatal sepsis, recent clinical studies claimed the need of other salivary markers for diagnosis due to unreliable screening validity of salivary CRP [18,19,40].

Because of this contradicting data basis for CRP as a salivary biomarker, we measured the CRP concentrations in saliva and serum samples of septic neonates to clarify the clinical relevance of CRP in this setting. As our clinical study showed a correlation between saliva and serum concentrations, especially for measurements taken over several timepoints, we decided to study the transport mechanism of CRP using in vitro models of the BSB.

For this purpose, we applied 10 µg/mL CRP in our oral mucosa and salivary gland model, as CRP concentrations of >8.7 µg/mL are associated with infections (together with high temperature), and clinically relevant concentrations of 10–20 µg/mL have also been used for studies with in vitro models of the blood–brain barrier previously [45,46]. While concentrations of 120 µg/mL CRP are measured in clinical settings in neonates [16], we focused on the cut-off concentration of CRP associated with infections to evaluate the diagnostic potential of CRP upon on-set of sepsis. Preliminary equilibrium studies with CRP in our oral mucosa model showed a reduced concentration in the basolateral compartment over 24 h, which was the first indication for a possible transport mechanism (Figure S1). Applying CRP in our oral mucosa and salivary gland epithelial models either on the apical (A/B) or basolateral side (B/A) (Figure 2) showed an elevated transport in the direction of saliva (B/A) in both models. In total, a transported average of 54 ng or 6.77 ng of CRP (molecular weight 118 kDa) was measured on the basolateral side after A/B transport in the oral mucosa and salivary gland model, respectively, while significantly higher amounts of CRP were detected in the apical compartment (oral mucosa: 112 ng; salivary gland model: 14 ng CRP) after B/A transport. These differences already confirmed the presence of directed transport processes for CRP in both models. To exclude that directed B/A transport is a compound-independent model intrinsic property, permeability studies with paracellular marker FD4 (molecular weight of 4 kDa) were accomplished (Figure S2). Data revealed no significant difference between FD4 transport from B/A and A/B in both models supporting that distinct transport mechanism for CRP are present in both models. Moreover, average P_app_ values of FD4 (oral mucosa model: 0.56-0.59 × 10^−6^ cm/s; salivary gland model: 0.29–0.33 × 10^−6^ cm/s) were concordant to the corresponding TEER values of the models and confirmed the stronger paracellular tightness of the salivary gland model.

Since literature data indicated a linkage between RAGE and CRP [31,47], we tested the expression of RAGE at the mRNA level of both models after CRP treatment resulting in effects on the expression pattern of RAGE similar to the permeability values of the transport studies with CRP (Figure 3A,B). 

This revealed that mRNA levels of RAGE are subject to regulation by CRP in an exposure side dependent manner. With regulation of RAGE after CRP treatment on the protein level, we decided to examine the possible involvement of RAGE in the transport of CRP. For this, we added soluble RAGE (sRAGE) to CRP for further transport studies.

In the oral mucosa model, this resulted in a significantly decreased permeability of CRP from B/A, while addition of sRAGE in the salivary gland model led to a significantly enhanced transport from B/A (Figure 4). In detail upon addition of sRAGE, a total transported average of 45.7 ng or 6.8 ng CRP was detected on the basolateral side after A/B transport in the oral mucosa and salivary gland model, respectively, while 74.6 ng (oral mucosa model) or 19.4 ng (salivary gland model) CRP was found on the apical side after B/A transport. Hence, addition of sRAGE elucidated RAGE-dependent CRP transport mechanisms in both models. Interestingly, mRNA expression of RAGE was not affected by addition of sRAGE to CRP in comparison to CRP alone in both models (data not shown) indicating that effects of sRAGE seemed to be restricted to the functional level of RAGE. In this context, it was shown that RAGE can mediate uptake as well as efflux transport processes [48,49]. In relation to our results, it could be speculated that RAGE mediates the uptake of CRP into saliva across the oral mucosa epithelium, whereas it is involved in efflux mechanisms to restrict exorbitant accumulation of CRP in saliva via the salivary gland epithelium.

Corresponding to the detected correlation of CRP in saliva and serum samples of neonates undergoing sepsis, both in vitro models of the BSB showed an elevated transport of CRP in the direction of saliva. While the favorable transport to saliva was shown in this study and the influence of RAGE on CRP transport was described, further studies regarding the underlying mechanisms would improve our current knowledge about the causal link between serum and salivary CRP and strengthen the relevance of CRP as a salivary biomarker in general. Future studies could aim at the identification of the responsible transporters, for example, by immunoprecipitation followed by mass analysis to screen for proteins binding to CRP in the cellular membrane fraction.

## 5. Conclusions

In this study on measurement of serum and salivary CRP levels, clinical data suggest that studying individual ratios and progression is more useful than relying on the measurement of a single time-point. Additionally, in vitro data using established models of the BSB show that CRP is preferentially transported to the direction of saliva, enhancing its feasibility as a salivary marker. Moreover, RAGE seems to be involved in the transport of CRP across the BSB. Future studies could focus on elucidation of the details of the transport mechanism of CRP across the BSB.

## Figures and Tables

**Table 1:** Applied primer sequences for 18SrRNA, Peptidylpropyl Isomerase A (PPIA) and Receptor for Advanced Glycation End Products (RAGE).

Primer	Forward 5′-3′	Reverse 5′-3′
18SrRNA	ATGGTTCCTTTGGTCGCTCG	GAGCTCACCGGGTTGGTTTT
PPIA	GTTCTTCGACATTGCCGTCG	TGAAGTCACCACCCTGACAC
RAGE	GAAGCTTGGAAGGTCCTGTCTC	CCGGAAAATCCCCTCATCCTG

**Table 2:** Characteristics of neonates at test with corresponding serum C-reactive protein (CRP) concentrations shown as mean ± SD (minimum (min.)–maximum (max.)) or numbers (*n*) and percentage (%).

Characteristics (*n* = 33 in 18 Patients)	Mean ± SD (Min.–Max.)/*n* (%)
Gestational age at birth (w)	33.1 ± 5.44 (25.4–40.6)
Corrected age at test (w)	38.4 ± 7.6 (28.4–55.4)
Day of life at test (d)	33.3 ± 44.7 (1–143)
Birth weight (g)	2170 ± 1130 (580–4400)
Body weight at test (g)	2870 ± 1330 (790–5040)
Female patients (*n*)	6 (33%)
CRP > 15 µg/mL at test (*n*)	22 (66%)

w—week; d—day; g—gram; *n*—number.

**Table 3:** Characteristics of healthy neonates without corresponding serum CRP concentrations shown as mean ± SD (minimum (min.)–maximum (max.)) or numbers (*n*) and percentage (%).

Characteristics (*n* = 22)	Mean ± SD (Min.–Max.)/*n* (%)
Gestational age at birth (w)	38.6 ± 1.6 (35.7–40.9)
Corrected age at test (w)	39.0 ± 1.6 (36.6–41.6)
Day of life at test (d)	2.8 ± 1.5 (1–6)
Birth weight (g)	3190 ± 610 (2090–4090)
Female (*n*)	11 (50%)
CRP > 15 µg/mL at test (*n*)	0 (0%)

w—week; d—day; g—gram; *n*—number.

## Data Availability

The data presented in this study are available on request from the corresponding author. The data are not publicly available due to privacy or ethical reasons.

## Supplementary Materials

The following are available online at https://www.mdpi.com/1999-4923/13/2/256/s1, Figure S1: Measured CRP concentrations in equilibrium studies at 2, 6, and 24 h using 10 μg/mL CRP in HBSS with the oral mucosa model based on TR146 cells. Values were compared to concentrations measured after 2 h and shown as % as mean ± SD with N = 7–8 of three independent experiments. Statistical analysis was performed with one-way ANOVA with post-hoc Holm-Sidak test, α = 0.05, *p* < 0.001 ***; Figure S2: Apparent permeability (Papp) for FD4 after 24 h from the apical to the basolateral side (A/B) and from the basolateral to the apical (B/A) side. Values shown as mean ± SEM (N = 4). Statistical analysis was performed as Student’s *t*-test with, α = 0.05 with *p* = 0.87 between A/B and B/A for the oral mucosa model and *p* = 0.41 between A/B and B/A for the salivary gland model.
