# Supplementary material for: Directed Transport of CRP Across In Vitro Models of the Blood-Saliva Barrier Strengthens the Feasibility of Salivary CRP as Biomarker for Neonatal Sepsis
Source: Pharmaceutics. 2021 Feb 12;13(2):256. doi: 10.3390/pharmaceutics13020256 (PMC7917918; doi:10.3390/pharmaceutics13020256)
Supplement: Supplementary file 1 [file pharmaceutics-13-00256-s001.pdf]

pharmaceutics

Supplementary materials: Directed Transport of CRP Across In Vitro Models of the Blood-Saliva Barrier Strengthens the Feasibility of Salivary CRP as Biomarker for Neonatal Sepsis

Grace C. Lin, Erik Küng, Merima Smajlhodzic, Sandra Domazet, Heinz P. Friedl, Joachim Angerer, Lukas Wisgrill, Angelika Berger, Lynne Bingle, Johannes R. Peham and Winfried Neuhaus

1. Supplementary Methods

1.1. TEER (Transepithelial electrical resistance) measurement

For TEER measurements cells were equilibrated at room temperature for 30–45 min after media change. The electrode (WPI, Sarasota County, FL, US, STX2) was disinfected for up to 10 min in sterile EtOH and subsequently equilibrated for at least 10 min in the respective media. Resistance values were measured with the Voltohmmeter (Merck, Darmstadt, Germany, MERS00001) as Ohmic values. Mean values of respective blanks were subtracted from the cell values and multiplied with surface area of the 24-well ThinCerts to give resistance values as $\Omega \times cm^2$.

1.2. Equilibrium studies

TR146 of passage 13–33 were seeded for equilibrium studies and cultivated as described in Section 2.3 of the main manuscript. On the day of the experiment transepithelial electrical resistance (TEER) was measured in the cultivation media (490.22 ± 62.81 $\Omega \times cm^2$, n = 33 from three independent experiments, mean ± SEM) and cells were washed twice apically with 300 µL or basolaterally with 900 µL HBSS. After measuring TEER in HBSS resulting in TEER values of 364.90 ± 30.86 $\Omega \times cm^2$, n = 33 from three independent experiments, mean ± SEM), the cells were exposed to HBSS containing 10 µg/mL CRP on the apical and basolateral side. Samples were taken for analysis with ELISA after 2, 6 and 24 h (shown in Figure S1). For ELISA the samples were diluted 1:12,5000 in HBSS prior to diluting 1:2 in PBS containing 1% BSA and 20 mM EDTA (Merck, Darmstadt, Germany, 324503) as described in the main manuscript.

1.3. Permeability assay with FD4

FD4 (FD4, 4 kDa, Sigma-Aldrich, St. Louis, MO, USA, ultra-filtered 1 mM stock solution) was applied on the apical (300 µL, A/B transport) or basolateral (900 µL, B/A transport) compartment at a concentration of 10 µM in DMEM media for the oral mucosa model or 1 µM in McCoy media for the salivary gland model upon cultivation on 24-well Thincerts as described in the main manuscript. Samples were drawn after incubation at 37 °C in the incubator for 24 h and relative fluorescence units (RFU) were measured at 488/520 nm using the Enspire Multimode Plate Reader (PerkinElmer, Waltham, MA, US). Permeability coefficient (apparent permeability, Papp) was calculated as described in the method section of the main manuscript and shown in Figure S2.

2. Supplementary Results

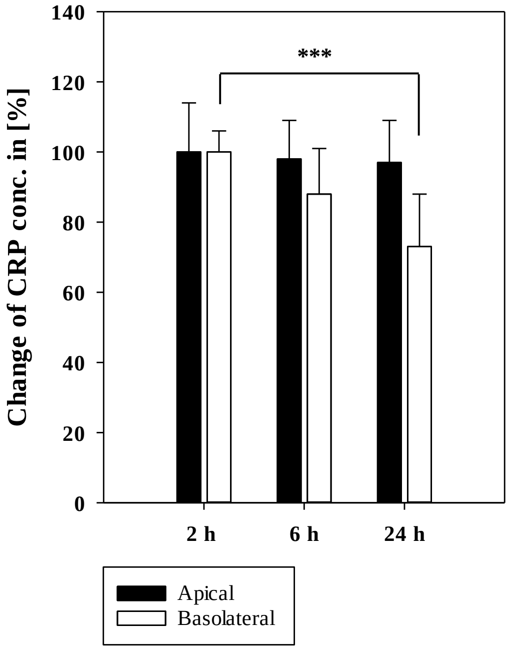

Figure S1. Measured CRP concentrations in equilibrium studies at 2, 6, and 24 h using 10 µg/mL CRP in HBSS with the oral mucosa model based on TR146 cells. Values were compared to concentrations measured after 2 h and shown as % as mean ± SD with n = 7–8 of three independent experiments. Statistical analysis was performed with one-way ANOVA with post-hoc Holm-Sidak test, $\alpha = 0.05$, $p < 0.001$***.

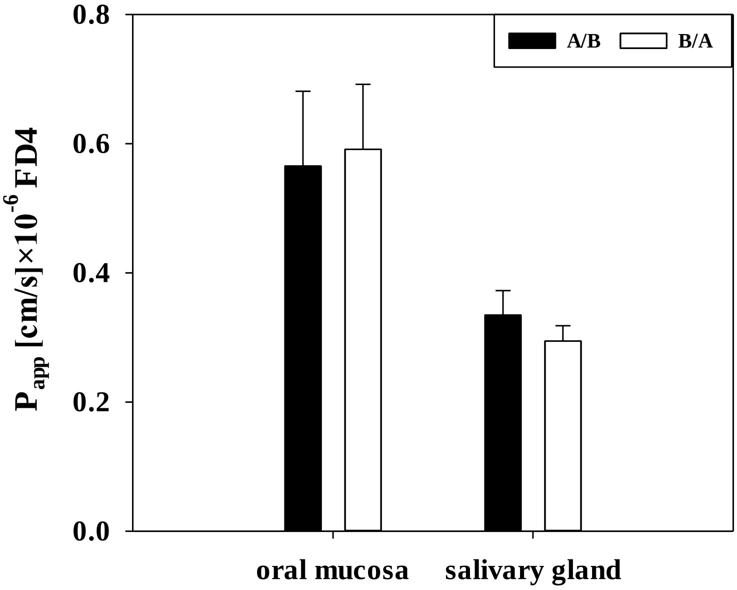

Figure S2. Apparent permeability (P_{app}) for FD4 after 24 h from the apical to the basolateral side (A/B) and from the basolateral to the apical (B/A) side. Values shown as mean ± SEM (n = 4). Statistical analysis was performed as Student´s t-test with, $\alpha = 0.05$ with $p = 0.87$ between A/B and B/A for the oral mucosa model and $p = 0.41$ between A/B and B/A for the salivary gland model.
